# Life Years Gained and Healthcare Dollars Saved: National Economic Evidence Supporting Comprehensive Genomic Profiling as Standard of Care for Canadian Cancer Patients

**DOI:** 10.3390/curroncol33040191

**Published:** 2026-03-30

**Authors:** Stephanie Snow, Shantanu Banerji, Yvonne Bombard, Don Husereau, Jason Karamchandani, Eddy Nason, Pamela S. Ohashi, Gijs van Rooijen, Gilad Vainer, Cassandra Macaulay, Filomena Servidio-Italiano

**Affiliations:** 1 Queen Elizabeth II Health Sciences Centre, Halifax, NS B3H 2Y9, Canada; stephanie.snow@nshealth.ca; 2 CancerCare Manitoba, Winnipeg, MB R3E 0V9, Canada; sbanerji@cancercare.mb.ca; 3 Institute of Health Policy, Management & Evaluation, University of Toronto, Toronto, ON M5T 3M6, Canada; yvonne.bombard@utoronto.ca; 4 Genomics Health Services Research Program, Li Ka Shing Knowledge Institute, St. Michael’s Hospital, Unity Health Toronto, Toronto, ON M5B 1T8, Canada; 5 School of Epidemiology and Public Health, University of Ottawa, Ottawa, ON K1N 6N5, Canada; dhuserea@uottawa.ca; 6 Department of Pathology, McGill University, Montreal, QC H3A 2B4, Canada; jason.karamchandani@mcgill.ca; 7 Sgnal49 Research, Ottawa, ON K1P 5J2, Canada; nason@signal49.ca; 8 Princess Margaret Cancer Centre, Department of Immunology, University of Toronto, Toronto, ON M5G 2C4, Canada; pam.ohashi@uhn.ca; 9 Genome Alberta, Calgary, AB T2L 2A6, Canada; info@genomealberta.ca; 10 Onco-Proteomics Lab, Pathology Department, Hadassah Medical Center, Jerusalem 91120, Israel; giladwv@gmail.com; 11 Colorectal Cancer Resource & Action Network (CCRAN), Toronto, ON M4W 3E2, Canada; cassandra.m@ccran.org

**Keywords:** cancer, comprehensive genomic profiling, costs and benefits analysis

## Abstract

Comprehensive genomic profiling (CGP) is a meaningful advancement in the field of oncology, enabling critical clinical decision-making regarding precision treatments that have biological rationale. In June 2025, the Colorectal Cancer Resource & Action Network (CCRAN) hosted their annual pan-tumour Biomarkers Conference, a virtual meeting of clinicians, scientists, and patients, to discuss recent progress in overcoming barriers to CGP access for patients in Canada with metastatic cancer. The meeting’s cornerstone was the presentation of the first national costs and benefits analysis of universal CGP for five metastatic tumour types; findings demonstrated this diagnostic’s potential, with the model estimating a gain of 3440 life years while generating $87M–134M of potential healthcare system savings, over a six-year time horizon. Additionally, conference sessions focused on the clinical value of CGP, strategies to leverage the economic analysis results and learn from international experiences, as well as mechanisms to prepare the Canadian healthcare system for future adoption. The conference led to calls to action for a national strategy to reduce disparities in equitable access to CGP, funding allocation for CGP as a standard of care for all patients with metastatic cancer, and pathways to enhance current infrastructure to expedite CGP across the country.

## Simple Summary

In 2025, the Colorectal Cancer Resource & Action Network (CCRAN)’s hosted their virtual Biomarkers Conference as a forum for clinicians, scientists, and patients to discuss the clinical value of comprehensive genomic profiling (CGP) for individuals with metastatic cancer and overcoming barriers to access. The keynote presentation was of the first national costs and benefits analysis of universal CGP for five metastatic tumour types; findings estimated a gain of 3440 life years and $87M–134M of potential healthcare system savings, over a six-year period. Conference sessions focused on strategies to leverage these results while learning from international experiences, in addition to mechanisms to facilitate CGP access in Canada. Several calls to action were recommended: a national strategy to reduce disparities in equitable CGP access, allocation of funding for CGP as a standard of care for all patients with metastatic cancer, and methods to enhance current infrastructure to facilitate progress.

## 1. Introduction

Cancer is the leading cause of mortality in Canada [1], and due to population growth and ageing, projections indicate that further increases in the total number of cases and deaths are imminent [2,3]. The implications for society at large and the healthcare system specifically are significant, as the economic burden of cancer in this country is already high ($26 billion in 2021) with 30% of costs borne by patients and their families (average of $33,000 per patient) [4]. Fortunately, treatments and innovations in cancer care have rapidly progressed over the recent decades, putting us in a stronger position to provide effective care for current and future cancer patients [5]. In particular, the cancer care landscape has been revolutionized by the introduction of comprehensive genomic profiling (CGP), where new platforms for molecular testing such as next-generation sequencing are utilized to examine the genomic sequence of tumours, simultaneously assessing up to hundreds of genes in a single test, and detecting the presence of specific biomarkers that drive tumour growth and spread [6]. In addition to somatic variants, CGP may also detect the presence of potential germline alterations which can be further investigated for validation [7]. The remarkable potential of CGP lies in its actionability; test results can guide individualized treatment decisions for patients with metastatic cancer, harnessing the potential for precision medicines and reducing exposure to costly, ineffective and toxic treatments [8,9]. As the field evolves, and additional novel biomarkers emerge for various tumour types [10,11,12,13,14], the clinical utility of CGP will further increase, progressing towards the goal of providing tailored, effective care for all patients with metastatic cancer.

The 2025 Biomarkers Conference is part of a Colorectal Cancer Resource & Action Network (CCRAN)-initiated annual, pan-tumour series that began in 2023 to address the delays in access to biomarker testing and results [15]. The latest meeting was an opportunity for patients, clinicians, pathologists, scientists, researchers, and policy-makers to discuss progress and learnings from the previous year (Supplementary Material), most notably the results of the national costs and benefits analysis of universal CGP in Canada. Conference panels also focused on the identification of policies and advocacy strategies to address key barriers to CGP use and facilitate Canada’s preparedness to implement CGP as a standard of care (SOC) for patients with metastatic cancer, to lead to more successful treatment, greater longevity, improved quality of life and decreased costs of care.

## 2. Economic Evidence Supports CGP as Standard of Care for Individuals with Metastatic Cancer

Although having CGP results early in the treatment journey can have a life-changing impact on patient outcomes, this testing is not currently SOC across all tumour types in any Canadian province or territory, and many patients with metastatic cancer face multiple personal and systemic access barriers [15]. During the conference, the results of CCRAN’s 2025 Canadian Clinician Pan-Tumour CGP Survey were shared; the online survey was developed to assess how medical oncologists across the country utilize CGP for patients with metastatic cancer, and was disseminated via the Canadian Association of Medical Oncologists (CAMO), as well as the social media channels of 23 collaborating patient organizations. The respondents comprised 40 medical oncologists across eight provinces, with the majority (70%) practicing out of academic centres. Although more than 80% of the respondents reported that CGP’s greatest benefits are its ability to identify additional personalized treatment options, including potential off-label therapies, and or appropriate clinical trials, close to two-thirds of respondents indicated that they were unable to access CGP for over 80% of their metastatic patients. Multiple barriers continue to limit clinical implementation across tumour types, corroborating the results of CCRAN’s 2024 National Pan-Tumour Patient Survey, a collaboration of 24 cancer patient organizations, to understand lived experiences with obtaining advanced diagnostic testing. The online survey was disseminated to potential participants (metastatic patients and caregivers) across Canada via patient organization outreach including email blasts and social media channels. Based on the findings from 183 respondents, specific CGP access challenges included a lack of awareness on the importance of testing, delays and lack of standardization in testing and obtaining test results, and inconsistent clinician engagement in discussions about CGP [16]. Although the generalizability of the results of the two above-mentioned surveys may have been impacted by their small sample sizes, as well as non-response bias, their collective findings revealed that the primary barrier preventing equitable CGP access for patients with metastatic cancer was lack of funding across tumour types and provinces. It was recognized that addressing this barrier through publicly funded coverage could only be feasible with robust national economic data to support programming, thus providing the inspiration to generate and present this data for the 2025 Biomarkers Conference.

### 2.1. Findings from the National Costs and Benefits Analysis of CGP as SOC

**Figure 1:**
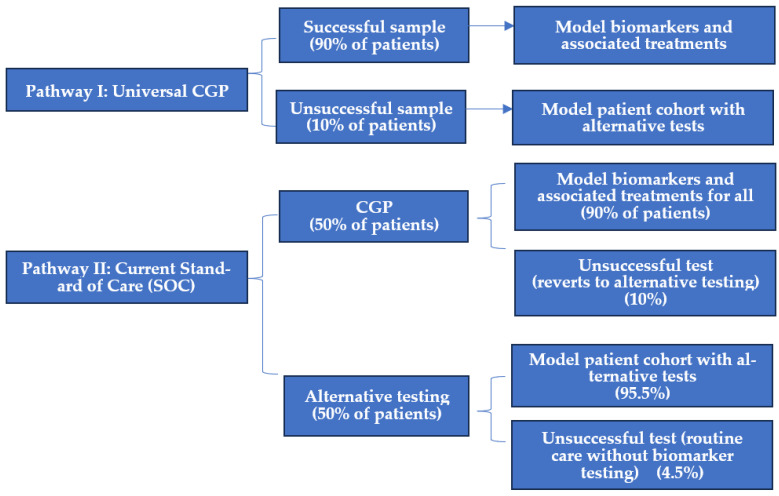
Framework of national costs and benefits model for CGP vs. current SOC.

In 2024, CCRAN engaged The Conference Board of Canada (now known as Signal49 Research) to model and analyze the costs and benefits of universal CGP for the five tumour types with the highest national age-standardized mortality rates (lung, colorectal, pancreas, breast, and prostate) over a six-year time horizon (2025–2030), assuming a societal perspective [17]. The study objective was to understand the economic and health impacts of two pathways for de novo Stage IV diagnoses for each of these five cancers, which together account for over 50% of Canadian cancer-related deaths [18]. Cancer incidence rates were estimated based on historical age-standardized incidence rates from 1995 to 2024, projected to 2030. Pathway I employed a universal model in which all patients received one of four different tissue-based CGP panels, ranging in size from 50 to 324 genes, and selected due to their current use in Canada as well as public data availability (Figure 1). Pathway II reflected the current SOC, a 50:50 mix of publicly funded CGP and alternative testing (e.g., sequential single-gene exclusionary, non-comprehensive, or rapid panel testing). For each pathway, the model applied incidence rates of both established and emerging biomarkers with demonstrated clinical utility to estimate patient eligibility for treatment. Up to four different treatment regimens were assigned per biomarker, reflecting Canada-specific available options. Total costs per pathway comprised treatment costs, panel costs (CGP or alternative panels), delayed care costs associated with the turnaround times for testing type, and alternative testing costs for those with unsuccessful CGP tests. Modelled benefits included both life years gained (LYG) due to identifying biomarkers with companion therapies, and societal contribution due to increased total income linked to improved patient outcomes. LYG was calculated separately for each scenario, based on the rate of actionable biomarker detection, the rate of matched therapy administration, as well as overall survival rate. Because of the limited evidence for the other cancer types, the following NSCLC data were used as proxies, given that robust real-world data were available for both CGP and sequential testing [17].

- Actionable biomarker identification rate for CGP: 32% [19];
- Matched therapy administration rate for CGP when a biomarker is identified: 43% [19];
- Actionable biomarker identification rate for alternative testing: 14% [19];
- Matched therapy administration rate for alternative testing when a biomarker is identified: 38% [19];
- Increase in overall survival from receiving CGP: eight months [19].

To measure the societal contributions of individuals, total income was calculated, comprising employment income for a percentage of the cohort (recognizing that many patients would not be working while in the late stage of cancer), investment income, private retirement income, other regular cash income, and government transfers (e.g., employment insurance) [20]. The median total income during the time horizon was calculated, while adjusting for inflation [21].

The analysis found that for all but the largest panel, universal CGP was less costly overall than the current mixed SOC. The cost-savings of using CGP over the mixed approach varied by cancer and by test, but resulted in cost-savings of $715 to 2495 per patient, on average (Table 1), for a total of $87M–134M of potential healthcare system savings.

The findings demonstrated that the use of CGP for these five metastatic tumours would result in 3440 LYG (Table 2) and a societal monetary return of $180 million. Cost reduction with universal CGP versus current SOC was demonstrated for all five metastatic cancers included in the economic model, with the most substantial savings observed for colorectal, pancreatic, and lung cancers, tumour types with the highest number of new metastatic cases. It is important to note that even with the largest CGP panel, the incremental cost of the CGP pathway compared to SOC was fairly minimal, increasing the cost by only $131 to $770 per patient, across the five tumour types. One of the primary mechanisms in which univeral CGP reduces cost compared to SOC is by providing comprehensive tumour biomarker information that informs a personalized treatment plan, often eliminating the initial use of costly yet ineffective therapeutics.

The results of the costs and benefits analysis demonstrated that the univeral implementation of CGP across Canada for patients with metastatic cancer could result in considerable survival benefits for patients at decreased cost compared to the current SOC. These results support and expand upon economic evidence for non-small cell lung cancer (NSCLC) generated by both real-world patient data in the United States and Germany demonstrating that the use of CGP is cost-effective versus small panel testing [22], and a Canadian budget impact model totalling the considerable non-material costs associated with single-gene testing [23]. The lower system costs observed in the 2025 Canadian model’s universal CGP pathway was primarily driven by reduced testing delays and elimination of sequential testing [17]. In the SOC pathway, the series of sequential biomarker testing enables costs (and associated treatment delays which also contribute substantial costs to the healthcare system) to build, while the higher upfront costs of CGP panels are balanced by the simultaneous biomarker testing and single set of results, thereby reducing delays in diagnosis and facilitating earlier access to treatment.

Conference discussion regarding the analysis noted the real-world applicability of the modelled scenarios and assumptions, as well as the following limitations:

- Due to the lack of Canadian data regarding the clinical utility and application of CGP for de novo Stage IV cancers, related assumptions were based on a combination of peer-reviewed data (where available) and expert opinion. Data for NSCLC was used as a proxy for other cancer types where insufficient data were available to compare the rate of identifying actionable biomarkers, receiving matched therapies, and overall survival between CGP-NGS and current single biomarker testing for each stage IV disease and panel type.
- Treatment costs reflect publicly available data rather than real-world hospital pricing that may have included possible discounts.
- Estimates on the proportion of patients receiving each therapy, and subsequent attrition, were derived from expert opinion (oncologists from each cancer specialty), which may have created inconsistencies due to varying treatment choices across Canadian provinces and territories.

#### Long-Term Medical and Economic Value of CGP as SOC

While the costs and benefits analysis results alone indicate great promise for the implementation of CGP as SOC for all patients with metastatic cancer in Canada, the results are based on a modelled simulation, and subject to the limitations and accuracies of the assumptions and inputs. Conference discussion emphasized the need for real-world evidence to validate these findings, which is the focus of new initiatives being conducted by CCRAN to support the introduction of CGP in the Canadian cancer landscape. Such evidence will capture a more complete understanding of the outcomes, encompassing both the impact of changes in the parameter values, as well as medical and economic benefits that were not modelled but are anticipated with widespread CGP implementation:

- Data-driven health systems will generate evidence for clinical and research use [24], and realize the potential of large CGP panels through the identification of other actionable mutations, accelerating the increase in CGP’s clinical utility for tumour types for which CGP is not currently cost-effective.
- Utility will further increase as (i) novel biomarkers emerge, particularly for cancers where CGP previously had limited value, and (ii) indications for existing targeted therapies expand to include new tumour types.
- Identification of specific patient populations for whom potentially toxic suboptimal “standard” therapies offer little benefit will avoid healthcare system funds being inappropriately spent on the “one size fits all” approach to prescribing that exists when CGP testing is unavailable altogether or subject to access delays.
- Global clinical trials will be more likely launched in Canadian sites, generating more data and opportunity, and benefiting the cancer care ecosystem by shortening drug development times and reducing drug costs [25,26,27].
- Population-level CGP data within the diverse Canadian landscape population is likely to hold economic value for industry, directing future rounds of cancer therapy research and development.

Conference speakers also discussed the critically important reduction in current CGP access inequities across Canada that will result from making this diagnostic tool SOC. Furthermore, CGP can help reach populations with lower screening rates; by identifying hereditary cancer genes, the results may facilitate a more targeted screening process among relatives for early cancer detection. Finally, more Canadian clinical trial sites will reduce current access inequities in which only patients with the means and ability to travel or relocate to participate in international trials are able to enrol [28,29].

## 3. Advancing Towards CGP as Standard of Care

Given the results of the CGP costs and benefits analysis, many conference sessions focused on Canada’s current level of preparedness to implement CGP as SOC for individuals with metastatic cancer.

### 3.1. Canada’s State of Readiness for Implementation of Genome-Based Testing Programmes

In 2025, Don Husereau conducted an update to his 2023 state of readiness assessment [30] to determine how prepared Canada was to consider and adopt genetic- and genome-based testing programmes such as CGP, based on existing infrastructure, operations, and healthcare environment conditions [31]. Overall, Canada appears to be making progress and is partially ready for a future of genetic and genomic testing in medicine. The report also reveals modest additional progress has been made since 2023. All provinces examined in 2023 (British Columbia, Alberta, Ontario, Quebec, Nova Scotia) made some improvement, with the most notable upgrades in Ontario and Nova Scotia. Prince Edward Island and Canada’s Territories refer to other provinces and were not evaluated. Ontario’s progress is due to the establishment of a provincial genetics program (PGP), which acts as an established link to the Ontario Pathology and Laboratory program.

The report showed that there are still gaps in how new testing proposals are considered and evaluated (i.e., HTA approaches), finance approaches used (i.e., clear funding formulas and financing for test development), linked information systems required, and the need for more systemic educational approaches and resources to support the use of testing, informed by overarching educational strategies. It also revealed that all provinces are poor at engaging with patient organizations and private life science stakeholders.

In 2025, Canada’s Drug Agency (CDA) assembled an advisory panel to develop a consensus-based framework to assess the use of biomarker testing in oncology [32]. After incorporating stakeholder feedback, the final version will be disseminated to provincial and territorial governments across Canada and will be an important tool in informing critical decisions on the funding of molecular, genetic, and genomic biomarker testing in cancer care.

#### International Experiences with Adoption of CGP

International panellists described CGP utilization and access in their countries, highlighting recognition of the significant benefits of CGP testing globally. In Belgium, CGP is available nationwide through in-hospital testing for patients with advanced cancer; studies have demonstrated that 93% of cases were successfully sequenced, with actionable mutations identified in 81% of patients, a rate considerably higher than the 21% using nationally reimbursed, small panels [33]. In Israel, CGP has been found to be more economical than a series of single gene tests, and is currently provided for 18 different oncological indications. To shorten the time from diagnosis to treatment, CGP is currently provided for patients with metastatic cancers including CRC, prostate and triple-negative breast cancer, as well as patients with NSCLC at any stage. Across Australia, there is considerable variation in access to CGP, which is not currently a part of routine cancer care. However, patients with incurable or advanced cancer can access CGP at no cost through the PrOSPeCT (Precision Oncology Screening Platform Enabling Clinical Trials) programme offered by Omico, a national independent not-for-profit cancer organization. Omico’s Molecular Oncology Board (MOB) reviews each patient’s CGP results and provides a report to the referring clinician, including a list of clinical trials for which they may be eligible [34]. In the United States (US), ten drug therapies have tumour-agnostic approval and CGP is available for metastatic cancers through public and private insurance, in addition to free provision by the diagnostic companies. However, uptake of testing is suboptimal in the US, potentially due to lack of awareness among some clinicians that testing is accessible and concerns regarding being able to correctly interpret test findings, speaking to the importance of education.

### 3.2. Facilitators of CGP Program Implementation

Throughout the conference, speakers identified multiple strategies to facilitate Canada in the adoption of CGP as SOC for patients with metastatic cancer.

#### 3.2.1. Enhancing Clinician Awareness and Knowledge of CGP

As the evolution of precision medicine continues, and additional biomarkers emerge across tumour types, ensuring clinicians in the field of oncology are knowledgeable about these advancements is paramount. Enhanced genomics training is needed for those that procure the diagnostic tissue and order the testing (e.g., surgeons, gastroenterologists and respirologists), as well as for diagnostic and molecular pathologists and medical oncologists to ensure that they are able to accurately interpret complex results. While ordering CGP tests and interpreting their findings are not within the scope of responsibilities for primary care providers, they carry an important role in counselling patients and providing guidance for their discussions with their oncologists. To facilitate decision-making based on collective clinician knowledge on CGP, multidisciplinary molecular tumour boards are invaluable, allowing patient results to be reviewed by multiple experts who then make a consensus recommendation for optimal treatment plans, including clinical trial opportunities [35].

#### 3.2.2. Expedited Turnaround Time for CGP Results

The turnaround time of diagnostic test results is one of multiple laboratory-related capacity constraints [16], and is particularly critical for patients who have had CGP. It is imperative that CGP results are available in a timely manner to inform patient treatment plans, given that even slight delays may impact patient fitness for therapy and thus survival, as well as system and health costs; prior studies demonstrate that each week of delayed treatment for a patient with lung cancer costs the healthcare system $400 [36], and for patients with NSCLC, 4% would die during a therapy delay of one week [37]. Currently throughout Canada, laboratory staffing shortages for medical laboratory assistants and technicians (MLA/Ts), medical laboratory technologists (MLTs) and pathologists which have to be addressed to facilitate expanded use of CGP [38,39]. Proactively promoting technologist and laboratory-focused career paths to high-school and college students may be a valuable way of exposing the future Canadian workforce to these positions. In order to relieve the current work burden, laboratories must focus on efficiency and efforts supported by the utility of single-test larger panels. While balancing the cost increase may be facilitated through available options such as grants or philanthropic funding and the phase-out of tests of lower utility, the primary funding source should be provincial and territorial healthcare budgets, in order to standardize both testing quality and access across Canada.

Recognizing the vast geography of Canada, jurisdictions with centralized CGP testing face hurdles in transporting the tissue sample to the main location, with turnaround time from biopsy to results extended for samples originating from remote communities. Innovative solutions addressing these types of barriers may help reduce geographic inequities, such as the use of liquid biopsy in settings where tissue sampling carries significant challenges.

#### 3.2.3. Incorporating Partnerships into Decision-Making Towards Implementation

Conference speakers discussed the use of public–private partnerships (PPP) as a mechanism for advancing the implementation of CGP across provinces and territories. While challenges to this model exist including the need to create transparency around role definition, ownership and data-sharing, and responsibly managing the balance between patient-centric and commercial priorities, key benefits were noted such as alignment of stakeholder priorities, expertise and resource sharing, and shortening time from discovery to practice. Several PPP approaches to funding and implementing innovation in genome projects were presented:

- The Alberta Diagnostic Ecosystem Platform for Translation (ADEPT) is a translational research programme that provides clinical samples and related data to commercial innovators of diagnostic tools and platforms [40]. While programmes such as ADEPT do not guarantee such laboratory technology will have widespread adoption, they can support the generation of evidence needed to facilitate uptake. Additionally, other provinces can leverage findings.
- Genome Canada, an independent and federally funded not-for-profit organization, founded the Canadian Precision Health Initiative (CPHI), a PPP programme supported by the Government of Canada with investments from industry, academia and public sector partners [41]. The CHPI funded 12 foundation projects (including oncology projects) to generate large-scale data, mobilize and advance the utility of genomic health datasets.
- Healthy Outcomes Through Genomic Innovations, a Genome Alberta and Genome British Columbia programme focuses on PPP solutions to accelerate the equitable adoption of genomic testing into routine clinical care [42].

The pace of technological advancement is rapid, and PPPs may serve as a key facilitator in the creation of robust infrastructure for storing, accessing and sharing data, so that systems are flexible to adopt new groundbreaking innovations for patients with metastatic cancer.

## 4. Conclusions

The 2025 Biomarkers Conference served as the platform for the release of the results of the national CGP costs and benefits analysis, and the resultant multidisciplinary discussion regarding how to optimally prepare Canada’s healthcare system to expeditiously leverage these findings to support CGP becoming a SOC for all patients with metastatic cancer in Canada. The 2026 Conference theme will be advancements towards a national strategy for CGP in metastatic cancer care, based on progress in response to the recommendations that were generated from the 2025 meeting.

While there remain competing priorities in the healthcare system that have yet to be considered, implementing CGP as SOC has the potential to realize substantial economic and clinical benefits, and improving access can unlock those benefits for many more patients while continuing to reduce cost. The Biomarkers Conference participants developed the following key calls to action for policy leaders:

1. Collaboration must be facilitated between regulators, payers and industry to identify regional differences in current access to CGP, and to create a cohesive national strategy to implement CGP as SOC for all individuals with metastatic cancer. Inter-provincial and territorial efforts as well as the further development of PPPs will be critical to eliminate variation in access to and availability of timely CGP, enhance physician prescribing flexibility and share best practices in adopting innovation.
2. Funding should be allocated so that CGP is first publicly covered for the five tumour types that contribute to the highest cancer-related mortality (lung, CRC, pancreas, breast, and prostate). This initial investment will build the foundation within the public health system that will provide the proof-of-concept evidence needed to demonstrate the clinical utility of CGP, and serve as the impetus for program extension to other tumour types as emerging biomarkers are realized.
3. Investment in CGP education for oncologists and other healthcare providers is needed to ensure that these providers stay current on important advancements in precision medicine. Pathways include ongoing continuing medical education (CME) on how to interpret CGP results, facilitation of improved interprofessional collaboration between pathology and oncology professionals, and enacting on-site or virtual molecular tumour boards in cancer centres and regional hospitals to provide support and knowledge on patient cases.
4. Investment in laboratory medicine infrastructure and health human resources is critical to improving equitable and timely access to CGP and results.

## Figures and Tables

**Table 1:** Cost savings per patient between CGP and current SOC ^1,2^.

	FoundationOne CDx (324 Gene)	Oncomine Precision Assay (50 Gene)	AmpliSeq Focus Panel (52 Gene)	Oncomine Comprehensive Assay V3 (161 Gene)
Lung cancer	−$131	$1075	$933	$715
Colorectal cancer	−$306	$2495	$2138	$1677
Pancreatic cancer	−$730	$1751	$1560	$1161
Breast cancer	−$770	$272	$130	$4
Prostate cancer	−$639	$392	$250	$130

^1^ Cost inputs for tests: FoundationOne CDx = $2700; Oncomine Precision Assay = $1005.33; AmpliSeq Focus Panel = $1287.87; Oncomine Comprehensive Assay V3 = $1322. ^2^ Positive values indicate cost savings to the healthcare system.

**Table 2:** Life years gained comparison of CGP and current SOC.

	CGP	Current SOC
Lung cancer	5933	4114
Colorectal cancer	2503	1735
Pancreatic cancer	1342	930
Breast cancer	447	310
Prostate cancer	991	687

## Data Availability

No new data were created or analyzed in this study. Data sharing is not applicable to this article.

## Supplementary Materials

The following supporting information can be downloaded at: https://www.mdpi.com/article/10.3390/curroncol33040191/s1, Supplementary S1: Conference Organization; Supplementary S2: Collaborating Patient Advocacy Groups; Supplementary S3: Conference Registrants; Supplementary S4: Conference Agenda.
