# Supplementary material for: Life Years Gained and Healthcare Dollars Saved: National Economic Evidence Supporting Comprehensive Genomic Profiling as Standard of Care for Canadian Cancer Patients
Source: Curr Oncol. 2026 Mar 30;33(4):191. doi: 10.3390/curroncol33040191 (PMC13114272; doi:10.3390/curroncol33040191)
Supplement: Supplementary file 1 [file curroncol-33-00191-s001.zip › curroncol-4187219-supplementary.pdf]

Current Oncology

MDPI

Supplementary S1. Conference Organization

The 2025 Biomarkers Conference was organized by CCRAN, a national patient advocacy group which has broadened its mandate to encompass all tumour types, providing patient and caregiver support, education and advocacy in Canada.

The conference was a virtual event spanning two days. The objectives and agenda of the conference was overseen by an Expert Steering Committee comprised on the following members:

- Dr. Stephanie Snow (Queen Elizabeth II Health Sciences Centre, Halifax, NS, Canada) —Chair;
- Dr. Shantanu Banerji (CancerCare Manitoba, Winnipeg, MB, Canada);
- Dr. Yvonne Bombard (St. Michael's Hospital, Li Ka Shing Knowledge Institute, Unity Health Toronto & Institute of Health Policy, Management and Evaluation, University of Toronto, Toronto, ON, Canada);
- Dr. Kelvin Chan (Odette Cancer Centre, Sunnybrook Health Sciences Centre, Toronto, ON, Canada);
- Mr. Allan Chankowsky (Patient Expert; Stage 4 Salivary Duct Cancer Survivor)
- Ms. Laura Greer (Burson Canada, Toronto, ON, Canada);
- Dr. Mita Manna (Saskatchewan Cancer Centre, Saskatoon, SK, Canada);
- Dr. Brandon Sheffield (William Osler Health System, Brampton, ON, Canada); and
- Dr. David Thomas (The Centre for Molecular Oncology, University of New South Wales, Kensington, Australia)

Supplementary S2. Collaborating Patient Advocacy Groups

Table S1. Collaborating Patient Advocacy Groups and Partners

Patient Advocacy Groups	
AYA Canada	Leukemia & Lymphoma Society of Canda
BC Lung Foundation	Lung Cancer Canada
Canadian Breast Cancer Network	My Gut Feel Stomach Cancer Foundation of Canada
Brain Tumour Foundation of Canada	Myeloma Canada
Canadian Cancer Survivor Network	Pancreatic Cancer Canada
Canadian Organization for Rare Disorders	Prostate Cancer Foundation Canada
Cholangio-Hepatocellular Carcinoma Canada	Raymond Foundation
Coalition Priorité Cancer au Québec	Rethink Breast Cancer
Craig's Cause Pancreatic Cancer Society	Save Your Skin Foundation
Genomic Focus	The Cancer Collaborative
GI Cancers Alliance	The Walnut Foundation
GIST Sarcoma Life Raft Group Canada	
HPV Global Action	

Supplementary S3. Conference Registrants

The 2025 Biomarkers Conference included 604 registrants, representing various stakeholder groups, specifically healthcare professionals, patients, caregivers, industry partners, researchers, and policy-makers. Registrants were from Canada, United States, Tanzania, India, Germany, Nigeria, Indonesia, Pakistan, Ghana, Australia, and Hungary.

Supplementary S4. Conference Agenda

The meeting agenda is presented in Table 2. All sessions were held virtually.

Table S2. Conference Agenda

Session	Speakers
**Day 1:** Policy & Advocacy to Support the Implementation of Comprehensive Genomic Profiling for Patients with Metastatic Cancer in Canada<br><br>***Moderator:*** **Cassandra Macaulay,** Chief Research Officer, CCRAN	
Symposium Opening	**Filomena Servidio-Italiano,** President & CEO, CCRAN<br>**Dr. Lisa Dickson,** Stage 4 Colon Cancer Patient and Healthcare Provider
Collective Advocacy Efforts for Equitable & Timely Access to Advanced Biomarker Testing & Results for Metastatic Cancer Patients in Canada	**Cassandra Macaulay,** Chief Research Officer, CCRAN
Key Learnings from CCRAN's 2024 Biomarkers Conference	**Dr. Sharlene Gill,** Medical Oncologist, BC Cancer - Vancouver
Generating a Costs and Benefits Analysis to Help Support Access to Comprehensive Genomic Profiling (CGP) for Five Metastatic Cancers in Canada: Helping to Ensure CGP Becomes a Standard of Care in Canada!	**Eddy Nason,** Director of Health, Conference Board of Canada
Reacting to the Findings of CCRAN's Costs and Benefits Analysis Report: The Value of Accessing Comprehensive Genomic Profiling for Cancer Patients in Canada	***Moderator:***<br>**Filomena Servidio-Italiano,** President & CEO, CCRAN<br>***Panelists:***<br>**Jenn Gordon,** Lead Strategic Operations and Engagement, Rethink Breast Cancer<br>**Maureen Elliott,** Senior Manager, Programs and Support, Pancreatic Cancer Canada<br>**Bukun Adegbembo,** Director of Operations, Canadian Breast Cancer Network<br>**Stefanie Condon-Oldreive,** Founder and Director, Craig's Cause Pancreatic Cancer Society<br>**Winky Yau,** Manager, Medical Affairs, Lung Cancer Canada<br>**Austin Zimmer,** Support Services Manager & Research Coordinator, Prostate Cancer Foundation Canada
What Can We Learn from Early Adopters of Comprehensive Genomic Profiling?<br>An International Discussion	***Moderator:***<br>**Laura Greer**, Patient Expert; Senior Vice President and National Practice Lead, Health & Wellness, Burson Canada; Breast Cancer Advocate<br>***Panelists:***<br>**Prof. David Thomas,** Director, UNSW Centre for Molecular Oncology<br>**Dr. Gilad W. Vainer,** Onco-Proteomics Lab, Pathology Department Head, Hadassah Medical Center<br>**Dr. Razelle Kurzrock,** Professor of Medicine, Associate Director, Clinical Research, Linda T. and John A. Mellowes Chair, Precision Oncology, MCW Cancer Center
The State of Readiness Progress Report 2.0: How Have Canadian Health Systems Improved?	**Don Husereau,** Adjunct Professor of Medicine, University of Ottawa
What Will it Take to Implement Comprehensive Genomic Profiling as a Standard of Care in Canada for the Management of Metastatic Disease in Cancer Patients?	***Moderator:***<br>**Cassandra Macaulay,** Chief Research Officer, CCRAN<br>***Panelists:***<br>***Patient:***<br>**Laura Greer**, Patient Expert; Senior Vice President and National

	Practice Lead, Health & Wellness, Burson Canada; Breast Cancer Advocate<br>***Panelists:***<br>**Dr. Laura Weeks,** Director, Health Technology Assessment, CDA<br>**Don Husereau,** Adjunct Professor of Medicine, University of Ottawa<br>**Dr. Monika Slovinec D'Angelo,** Health System and Policy Consultant, Adjunct Professor, University of Ottawa<br>**Dr. David Stewart,** Medical Oncologist, The Ottawa Hospital - Cancer Centre; Professor of Medicine, University of Ottawa<br>**Dr. David Cescon,** Medical Oncologist & Clinician Scientist, Princess Margaret Cancer Centre; Associate Professor, Department of Medicine, University of Toronto<br>**Dr. Robert Bell,** Former Ontario Deputy Minister of Health; Professor Emeritus, Department of Surgery, University of Toronto
**Day 2: The Future of Comprehensive Genomic Profiling & Precision Medicine in Clinical Practice**<br>***Moderator:*** **Cassandra Macaulay,** Chief Research Officer, CCRAN	
Welcome from CCRAN's President	**Filomena Servidio-Italiano,** President & CEO, CCRAN<br>**Dr. Christine Qiong Wu,** Stage IV Cancer Patient; Professor of Engineering, University of Manitoba
Reviewing the Findings of a National Canadian Clinician Survey: Identifying Current Clinical Utilization & Utility of Comprehensive Genomic Profiling for Patients Diagnosed with Metastatic Cancer Across Multiple Tumour Types	**Shalini Gambhir,** Research Officer, CCRAN<br>**Cassandra Macaulay,** Chief Research Officer, CCRAN
It Starts at the Lab: Towards Establishing National Standards for the Performance, Integration, Coordination and Communication of Biomarker Testing Results	***Moderator:***<br>**Dr. Shantanu Banerji,** Director of Precision Oncology and Advanced Therapeutics, CancerCare Manitoba<br>***Patient:***<br>**Robby Spring,** Patient Advocate; Breast Cancer, Luminal B, Stage 1 Survivor<br>***Panelists:***<br>**Dr. Doha Itani,** Associate Professor, Anatomic & Molecular Pathology, Dalhousie University; Division Head, Molecular Diagnostics & Cytogenetics, Saint John Regional Hospital<br>**Dr. Jason Karamchandani,** Associate Professor, Departments of Pathology, Neurology and Neurosurgery, McGill University; President, Canadian Association of Pathologists<br>**Dr. Angela Hyde,** Medical Oncologist & Clinician Scientist, NL Health Services<br>**Dr. Janessa Laskin,** Medical Oncologist and Clinical Researcher, BC Cancer
Why Access Comprehensive Genomic Profiling?<br>The Many Promising Therapeutic Benefits of a Genome-First Approach	***Moderator:***<br>**Dr. Thejus Jayakrishnan,** Medical Oncologist, Dana-Farber Cancer Institute and Brigham and Women's Hospital; Instructor in Medicine, Harvard Medical School<br>***Patient:***<br>**Matt Reidy,** Founder, Genomic Focus; Long-term, Stage 4 Cancer Survivor<br>***Clinician Roundtable:***<br>**Dr. Michael Raphael,** Medical Oncologist, Odette Cancer Centre, Sunnybrook Health Sciences Centre<br>**Dr. Mita Manna,** Medical Oncologist, Saskatoon Cancer Centre; Associate Professor, Department of Oncology, University of Saskatchewan<br>**Dr. Ravi Ramjeesingh,** Medical Oncologist & Assistant Professor, Chair of the HPB Cancer Disease Site Group Halifax, Division of

	Medical Oncology and Department of Community Health and Epidemiology, Nova Scotia Cancer Centre & Dalhousie University<br>**Dr. Stephanie Snow,** Medical Oncologist, QEII Health Sciences Centre; Professor, Dalhousie University; President, Lung Cancer Canada<br>**Dr. Shantanu Banerji,** Director, Precision Oncology and Advanced Therapeutics, CancerCare Manitoba<br>**Dr. Laura Hopkins,** Gynecologic Oncologist, Saskatchewan Cancer Agency; Professor in the Department of Oncology, University of Saskatchewan's College of Medicine
Activating the Immune Response: Exploring Novel Avenues of Immunotherapy	***Moderator:***<br>**Dr. Michael Raphael,** Medical Oncologist, Odette Cancer Centre, Sunnybrook Health Sciences Centre<br>***Caregiver:***<br>**Nora Woo,** Caregiver of Spouse who succumbed to Stage IV Colorectal Cancer; Patient Advocate<br>***Panelists:***<br>**Dr. Pamela Ohashi,** Senior Scientist, Princess Margaret Hospital; Director, Tumor Immunotherapy Program; Professor, Department of Immunology, Faculty of Medicine, University of Toronto<br>**Dr. Megan Mahoney,** Director, Scientific Affairs and Training, BioCanRx
Understanding Variability in Genomic Testing Across Canada: An HCP Survey Presentation *(pre-recorded presentation delivered during a scheduled break)*	**Sabrina Hanna, Chief Change Officer, The Cancer Collaborative**
Innovative AI Based Technology in the Management of Metastatic Cancer	***Moderator:***<br>**Dr. Yvonne Bombard,** Canada Research Chair, Genomics Health Services & Policy; Professor, University of Toronto; Director & Scientist, St. Michael's Hospital; Co-Founder & CEO, Genetics Adviser<br>***Caregiver:***<br>**Chamundeeswari Srinivasan,** Caregiver; Patient Advocate; Healthcare Strategist<br>***Panelists:***<br>**Dr. Rob Grant,** Medical Oncologist, Princess Margaret Cancer Centre, University Health Network<br>**Dr. Omar Khan,** Clinical Assistant Professor, Medical Oncology, University of Calgary<br>**Dr. Trevor Pugh,** Associate Professor, Department of Medical Biophysics, University of Toronto; Lead, Clinical Genomics Program, Princess Margaret Cancer Centre; Senior Scientist, Princess Margaret Cancer Centre
Coming in 'Hot': The Future of Radioligand Therapies in Canada	**Dr. David Laidley,** Nuclear Medicine Oncologist; Associate Professor, Western University
The Value of Partnerships to Drive Innovation in Health Care Systems	**Gijs van Rooijen,** Chief Scientific Officer, Genome Alberta<br>**Étienne Richer,** Director of Genomics Programs, Genome Canada
Closing remarks	**Filomena Servidio-Italiano,** President & CEO, CCRAN
